# Comparison between Restorative Materials for Pulpotomised Deciduous Molars: A Randomized Clinical Study

**DOI:** 10.3390/children10020284

**Published:** 2023-02-01

**Authors:** Kanwalpreet Kaur, Bharat Suneja, Sunaina Jodhka, Ravinder S. Saini, Saurabh Chaturvedi, Shashit Shetty Bavabeedu, Fahad Hussain Alhamoudi, Marco Cicciù, Giuseppe Minervini

**Affiliations:** 1 The Dental Clinic, 92-Sant Nagar, Civil Lines, Ludhiana 141010, Punjab, India; 2 Department of Pediatric and Preventive Dentistry, Baba Jaswant Singh Hospital and Research Institute, Chandigarh Road, Ludhiana 141010, Punjab, India; 3 Dental Technology Department, COAMS, King Khalid University, Abha 62529, Saudi Arabia; 4 Department of Prosthetic Dentistry, College of Dentistry, King Khalid University, Abha 62529, Saudi Arabia; 5 Restorative Dental Sciences, King Khalid University College of Dentistry, Abha 62529, Saudi Arabia; 6 Dental Technology Department, Applied Medical Science, King Khalid University, Abha 62529, Saudi Arabia; 7 Department of Biomedical and Dental Sciences, Morphological and Functional images, University of Messina, G. Martino Polyclinic, 98158 Messina, Italy; 8 Multidisciplinary Department of Medical-Surgical and Odontostomatological Specialties, University of Campania “Luigi Vanvitelli”, 80138 Naples, Italy

**Keywords:** pulpotomy, stainless steel crowns, Cention-N, primary molars, children, pediatric

## Abstract

Aim: To evaluate and compare the clinical outcomes of Cention-N (CN) and stainless steel crowns (SSCs) as restorations for pulpotomised primary molars, and to study clinical and radiographic outcomes of pulpotomies restored with these materials. Methods: The study was conducted on 60 pulpotomised molars with occlusoproximal caries. These were randomly divided into two groups and restored with either stainless steel crowns or Cention-N. Clinical performance of restorations and clinical and radiographic success of pulpotomy was examined at 6, 9 and 12 months. Results: The mean scores for marginal integrity deteriorated significantly at 6, 9 and 12 months in both groups but in comparison were insignificant. The mean for proximal contact deteriorated significantly for the Cention-N group, whereas the mean for gingival health deteriorated remarkably for the stainless steel crown group at successive evaluations. No tooth in either group showed secondary caries or discomfort on biting, except for one tooth in Cention-N group which presented with secondary caries. The clinical success rate for pulpotomised molars was 100% for both groups until nine months, although this had reduced by the end of 12 months. Radiographically, the success rate was 79.3% for Cention-N, while it was 86.6% for stainless steel crowns at 12 months. There was no significant difference in clinical and radiographic success between either group. Conclusion: Cention-N and stainless steel crowns are comparable for marginal integrity. However, crowns maintain significantly better proximal contacts while Cention-N was notably better for gingival health of the restored tooth. Both materials do not show secondary caries and discomfort on biting and are comparable in clinical and radiographic success of pulpotomy at the end of one year.

## 1. Introduction

The procedure of pulpotomy involves the removal of the coronal portion of the pulp and is a recommended procedure for treating vital asymptomatic primary teeth with carious pulp exposures. The indications for pulpotomy are teeth with extensive caries, no spontaneous pain, no evidence of radicular pathology, reversible pulpitis and carious pulp exposure, and teeth that suffered trauma exposing the pulp [1]. However, the correlation between symptoms and pulpal status is frequently a challenging task for the paediatric dentist. Clinically, a pulpotomy is usually indicated once the breakdown of the marginal ridge is significant. The reason is that upon a proximal carious attack, pulpal inflammation in primary molars develops at an early stage and by the time proximal caries manifests itself clinically, the pulpal inflammation is quite advanced. Such teeth are indicated for a pulpotomy, and the removal of the coronal pulp alone would render the tooth free of inflammation [1]. It is a procedure where the chances of failure are low. However, whenever a failure does occur, it is usually attributed to an important factor of leakage due to incomplete coverage of the tooth, which causes bacterial penetration from the salivary environment into the pulp through open dentinal tubules. For this reason, a well-sealed restoration is critical to the survival of a damaged pulpotomised tooth [2]. Therefore, the rationale for the selection of a post-pulpotomy restoration is based on its sealing ability, strength, esthetics, and its acceptability to the child and parent [3,4]. Stainless steel crowns (3M ESPE, SSC) have been considered the gold standard for restoring pulpotomised primary molars [5,6], due to various properties such as complete coverage, minimal technique sensitivity, minimal marginal leakage, and their lack of sensitivity to oral conditions during cementation [7,8].

However, the choice of dental material is also influenced by patient preference and in the pediatric population, as in adults, the demand for esthetics has grown equally [9]. Moreover, the parents are involved in clinical decision-making more than ever before and over the course of time, preferences of the parents, concerning the choice of dental materials for their children, have evolved based on their esthetic value, toxicity, cost, and durability [10,11,12,13]. Esthetic options, although available as full coverage crowns in primary molars, are not routinely recommended, either due to difficulties in placing these (pre-veneered or open-faced stainless steel crowns) or due to their higher cost (zirconia crowns) [14,15,16]. Likewise, esthetic SSC incorporating composite or porcelain veneers on the buccal side was introduced. Although these received parental approval, drawbacks such as shade mismatch, the tendency for veneer fracture, crimping inability, and lack of durability, limited their use [14]. Esthetic concerns have also been raised for open-faced SSC [17,18,19].

Intra-coronal materials like glass ionomers and resin-modified glass ionomer cement have also been evaluated as post-pulpotomy restorative materials. These have gained popularity and have given esthetically pleasing results in patients. However, these cements have certain disadvantages of low fracture and wear resistance. The introduction of composites conferred relatively better physical properties, marginal adaptation and esthetics, and they have been used for restoring pulpotomised primary molars [20]. Despite the advantages, the technique sensitivity, polymerization shrinkage, inadequate flexural strength, a longer placement time, and the need for the cooperation of a child dictate that this is not the material of choice [21].

Hence, it seems that it would be desirable to have an alternative which restores the tooth to its original strength, adequately prevents marginal leakage, is esthetically acceptable to the child as well as to the parent, and at the same time is gentle to the periodontal tissues. A new restorative material, Cention N (Ivoclar Vivadent; CN, Shanghai, China), which belongs to a group of alkasites, was introduced in October 2016. It offers tooth-colored esthetics, dual curing, minimal technique sensitivity and high flexural strength (more than 100 MPa), due to which it has been considered suitable for use in stress-bearing posterior regions [22]. CN releases fluoride and calcium ions favoring remineralization. Its translucency of 11% provides superior esthetics and the patented alkaline fillers (Isofillers) release hydroxide ions that regulate the pH during acid attacks, preventing demineralization. The fillers also relieve stress, lessening the shrinkage. Decreased shrinkage causes lesser microleakage in CN as compared with composites. The stable and efficient self-cure initiator in CN causes a high degree of polymerization due to the cross-linking of methacrylate monomers. Its initiator system also enables chemical curing and light curing, known as a dual cure. A study by Tiskaya assessed the ion release, pH changes and apatite formation ability of two potentially bioactive composites: Cention N (CN) and Activa (ACT). CN has bioactive properties that may explain the low incidence of secondary caries found clinically with this composite [23]. The results of a previous study on the effect of adding bioactive glasses in 3D printed material show that modification of methacrylate resin, used in 3D printing technology, with bioactive glasses produces novel dental materials that possess desirable bioactive properties [24].

Published literature in pediatric dentistry shows only limited research for ideal restorative materials to be used after the procedure of pulpotomy. There is voluminous literature available on the choice of pulpotomy agent, however, there has been considerably less scrutiny on the type of post-pulpotomy restoration. The clinical success of an alkasite material (Cention-N) being used for post-pulpotomy restoration in primary molars has not been reported in the literature. Therefore, this study was conducted with the aim to evaluate the clinical performance of an alkasite material (Cention-N) as compared to stainless steel crowns as a restorative material in pulpotomised primary molars at 6, 9 and 12 months, and to compare the clinical and radiographic success of pulpotomised molar when restored with either of these materials at the end of 12 months. The null hypothesis formulated was that there will be no variation in the clinical and radiographic success of pulpotomised molar restored with alkasite material (Cention-N) or stainless steel crowns.

## 2. Materials and Methods

### 2.1. Study Design

This trial was designed after multiple discussions among the authors and strictly followed the guidelines of CONSORT (Consolidated standards of reporting trials) [25]. It was a single-centered and double-blinded randomized clinical trial, approved by an institutional ethical review board (Ethics Approval Number-BFUHS/2k19/p-TH). It was registered in the clinical trial registry in India with the number CTRI/2023/01/048831. All procedures, possible discomforts, risks and benefits were explained to the parents of the children involved and informed written consent was obtained from all.

Centralized computer-generated randomization was done without any restrictions. Randomization codes were kept sealed and managed by the central pharmacy to follow the allocation concealment. The treatment with study variables was performed by two blinded examiners, and associated recordings were done. Each time, while checking, a new sheet of paper was given to the examiners to record the measurements, and to hide the previous measurements, in order to prevent bias. Interventions were carried out by another blinded examiner. Cronbach’s Alpha for inter-examiner reliability was found to be 0.92 and intra-examiner reliability 0.85 and 0.82 for examiners 1 and 2, respectively.

### 2.2. Source and Method of Collection of Data

This randomized clinical trial was carried out on children aged 4–8 years, in the Department of Pediatric and Preventive Dentistry, Baba Jaswant Singh Dental College, Hospital and Research Institute, Chandigarh Road, Ludhiana, Punjab, India. The patients were screened, clinically as well as radiographically, for one or more carious primary molars (maxillary or mandibular), with two surface involvement (occluso-proximal) indicated for pulpotomy, as described in the inclusion criteria. The criteria were based on AAPD (American Academy of Pediatric Dentistry) guidelines 2009, which state that the pulpotomy procedure is performed when caries removal results in pulp exposure in a primary tooth with normal pulp, reversible pulpitis, or after traumatic pulp exposure. The decided inclusion criteria were: Clinically, an asymptomatic primary molar with an occluso-proximal carious lesion involving the marginal ridge (more than half of the marginal ridge, studied by measuring the intercuspal distance bucco–lingual) involved in the carious process [1]. The involved teeth should have no signs of pathologic mobility, no swelling or fistula, no history of spontaneous or nocturnal pain, and tenderness to percussion or palpation. Radiographically, a deep carious lesion approaching the pulp with no evidence of periapical pathologies such as an abscess, periodontal space widening, periapical or furcation radiolucency, or internal or external resorption. Teeth having no more than one-third of their roots undergoing physiologic resorption as evaluated by IOPAR (Intraoral periapical radiograph). Exclusion criteria: Children with any kind of systemic disease (developmental anomalies and compromised immunity); children with non-acceptable oral hygiene with a plaque index score of as high as three (abundance of soft deposits within the gingival pocket and/or on the gingival margin and adjacent tooth surface).

### 2.3. Distribution of Teeth in the Sample

In the CN group (Group-I): for the maxillary molars, there were six maxillary right first primary molars (54) accounting for 20%, seven maxillary right second primary molars (55) accounting for 23.3%, two maxillary left first primary molars (64) accounting for 6.7%, and two maxillary left second primary molars (65) accounting for 6.7%. For the mandibular molars, there were six mandibular left first primary molars (74) accounting for 20%, one mandibular left second primary molar (75) accounting for 3.3%, five mandibular right first primary molars (84) accounting for 20%, and one mandibular right second primary molar (85) accounting for 3.3%.

**Figure 1:**
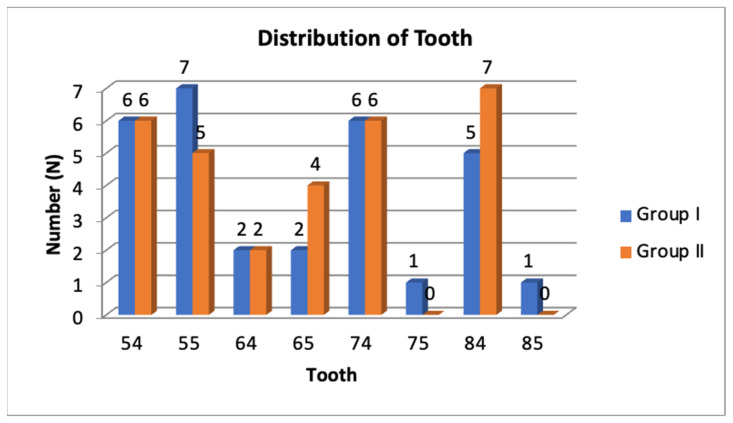
Distribution of teeth in the total sample.

In SSC group (Group-II): for the maxillary molars, there were six maxillary right first primary molars (54) accounting for 20%, five maxillary right second primary molars (55) accounting for 16.7%, two maxillary left first primary molars (64) accounting for 6.7%, and four maxillary left second primary molars (65) accounting for 13.3%. For the mandibular molars, there were six mandibular left first primary molars (74) accounting for 20%, no mandibular left second primary molars (75) accounting for 0%, seven mandibular right first primary molars (84) accounting for 23.3%, and no mandibular right second primary molars (85) accounting for 0% (Figure 1).

### 2.4. Sample Size

A total of 200 patients, in the age group of 4–8 years were screened, out of which, 90 patients met the inclusion criteria. Guardians of 70 patients consented to the treatment as a part of a research study, however, only 60 subjects reported for treatment. According to Edward and Mascha, for a sample to be statistically examined, there must not be less than 30 subjects [26]. Therefore, the total sample size was calculated to be 60 subjects. All the advantages and disadvantages of the procedures to be carried out were explained to each parent and informed consent was obtained. The study entailed randomly allocating the patients into two groups with the chit system. Those with the odd-numbered chit were assigned to group 1 and those with the even-numbered chit were assigned to group 2. Children with more than one carious primary molar were asked to pick the same number of chits as the number of molars involved, and the teeth were then grouped for treatment accordingly. All the teeth in both groups were treated under rubber dam isolation (Dental Dam, Coltène Whaledent, Langenau, Germany). Group 1: The teeth were restored with an alkasite restorative material (Cention-N) after the procedure of pulpotomy of the carious primary molar. Group 2: The teeth were restored with SSC after pulpotomy.

### 2.5. Procedure for Pulpotomy and Restorations

All pulpotomies were performed by the same operator under local anesthesia following a standard clinical practice. The technique used for pulpotomy was performed by a standard procedure, and ferric sulphate (ViscoStat, Ultradent, 505 West Ultradent Drive, South Jordan, UT 84095, USA) was used as the material of choice for performing pulpotomy [27].

Clinical status and periapical radiographs were reviewed for each carious molar indicated for pulpotomy before treatment. After the procedure of pulpotomy in group 2, the tooth was restored with SSC in a standardized way (Richard J Mathewson., Fundamentals of Pediatric Dentistry, 3rd edition) [28], while in group 1, the overlying chamber was filled with the alkasite material CN.

The mixing ratio for CN was one measuring scoop of powder and one drop of liquid (corresponding to a weight ratio of 4.6:1). The powder was mixed with the liquid on a paper pad until a homogeneous consistency was achieved (mixing time: 45–60 s). The working time was 3 min from the start of mixing. The material was applied to the cavity, carefully adapted and condensed and any occlusal excess was removed. The setting time (self-curing) was 5 min from the start of mixing. Fendermate sectional matrix (Directa AB; 194,22, Upplands Vasby Sweden), which has an attached wedge, was used for proper cervical sealing of the restoration.

### 2.6. Assessment of the Outcomes

The assessments for both the restorations as well as for the pulpotomies were conducted over a one-year period. Clinical assessment was done after 6 months, 9 months and 12 months, while the radiographic assessment was done after 12 months [29]. All the procedures were performed by the blinded examiners and the outcomes were assessed by an independent observer other than the examiners at 6, 9 and 12 months.

Clinical and Radiographic Assessment of the Restorations

The clinical evaluation of the two restorations was assessed using parameters of marginal integrity, proximal contact, gingival health, secondary caries, and discomfort on biting, as per the modified USPHS (United States Public Health Service) criteria [30]. A score was assigned for each of these parameters depending on its severity (Table 1). Clinical and radiographic assessments of the pulpotomised teeth [31] were performed using a scoring criterion, as described in Table 2 and Table 3.

### 2.7. Statistical Analysis 

The recorded data was compiled and entered into a spreadsheet computer program (Microsoft Excel 2010), and then exported to the data editor page of SPSS version 17 (SPSS Inc., Chicago, IL, USA). Descriptive statistics including computation of percentages, means and standard deviations were calculated. The statistical tests applied for the analysis were Pearson’s chi-square test (χ^2^), Independent sample *t*-test and Paired *t*-test. For all tests, the confidence interval and *p*-value were set at 95% and ≤0.05, respectively. Intrarater reliability was determined by the intraclass correlation coefficient.

## 3. Results

The results of the statistical evaluation of parameters for the clinical assessment of restorations are described in Table 4 and Table 5. Statistical evaluations of the clinical assessment of the pulpotomised molars are described in Table 6, and of the radiographic assessment of the molars are described in Table 7.

MI (Marginal Integrity) deteriorated progressively for both groups at all evaluation periods, although the comparisons between the mean scores of the two groups were found to be nonsignificant at six (*p* = 0.83), nine (*p* = 0.31) and twelve (*p* = 0.5) months, respectively. Although PC (Proximal Contact) deteriorated progressively at all evaluation periods for molars restored with CN, the deterioration ceased by the end of nine months for SSC restorations. Nevertheless, the comparisons between the mean scores of the groups were found to be significant at all evaluation periods (*p* = 0.001). GH (Gingival Health) was significantly better around teeth restored with CN. However, deterioration of GH was significant with respect to molars restored with SSC at all evaluation periods. On comparing the mean scores between the groups, the differences were found to be significant at all evaluation periods (*p* = 0.001). Intragroup comparisons of the above parameters revealed that there was a progressive breakdown of MI and PC, with no effect on GH, for molars restored with CN. For molars restored with SSCs, there was a progressive breakdown of MI and deterioration of GH, with no effect on PC.

The clinical success rate for the pulpotomised molars was 100% for both groups at six and nine months. No tooth presented with pain, TP, swelling or TM in these evaluation periods. However, this reduced to 79.3% and 86.6% for groups 1 and 2, respectively, by the end of 12 months. The intergroup comparisons for clinical success rates were found to be nonsignificant at 12 months. 

On completion of the study period at the end of 12 months, molars restored with CN and SSC showed radiographic success rates of 79.3% and 86.6%, respectively. Nonetheless, intergroup comparisons were found to be nonsignificant. Thus, the overall clinical and radiographic success rate of the study was 84%.

## 4. Discussion

An optimal seal between the tooth and the restoration after pulpotomy is a must for the success of the treatment. There is a positive association between the clinical status of the treatment and the quality of the restoration. A pulpotomy mainly fails when the marginal integrity of the restoration is compromised, which breaches the barrier to the bacteria from the oral environment [32]. Along with excellent physical and mechanical properties, the current scenario has placed esthetics as an important factor in the child and parental acceptance of restorations [33,34]. An ideal post-pulpotomy restoration should be esthetic and should mimic the natural teeth in color, translucency and surface texture for a longer period [10,35]. The SSCs, so far has been considered the gold standard and most reliable and durable restorative material for restoring pulpotomised primary molars [5,6]. Seemingly the only concerns for these crowns are esthetics and the effect on periodontal tissues [36,37]. To overcome the steel-colored appearance of SSCs, esthetic SSCs have been introduced, such as open-faced SSCs with a facial or buccal coating of composite and zirconia crowns. Although parent satisfaction has been reported with these, drawbacks such as difficulty in shade matching, the tendency for veneer structure to fracture and zirconias requiring significantly more time to prepare the tooth, have prevented their universal acceptance by dentists [14,18,38]. Tooth-colored intracoronal materials have also been introduced, such as glass ionomer cement and composites. Although glass ionomer cement has esthetic, adhesive, biocompatible, and anticariogenic properties, it lacks toughness and cannot withstand the high-stress concentrations that promote crack propagation [20]. Despite the widespread acceptance of composite resins as being the conventional treatment in primary teeth with vital pulp therapy, there is limited evidence that this approach is the best option in the field of pediatric dentistry [39,40,41,42]. The main problem faced by pediatric practitioners using these adhesive restorations is technique sensitivity and absolute isolation with rubber dam [43], risk of microleakage [44], and subsequent secondary caries [22].

Therefore, an alternative post-pulpotomy restoration was used in the present study, which could possibly overcome the limitations posed by resin-based materials, as well as full coronal restorations. The results of the study show that there was no significant difference in clinical and radiographic success between either of the studied post-pulpotomy restorations, SSCs and CN, thus the null hypothesis was accepted. A new restorative material belonging to a group of alkasites was introduced in October 2016, offering excellent esthetics along with high flexural strength. According to the manufacturer, Cention N is a tooth-colored filling material with a high translucency of 11%, allowing it to blend in naturally with the surrounding tooth structure while covering discolored dentin at the same time. Clinical studies have confirmed that flexural strength of ≥100 MPa is an important factor for the stress-bearing posterior region in class 2 cavities [23]. Due to the use of cross-linking methacrylate monomers with a stable, efficient self-cure initiator, CN exhibits a high degree of polymerization and a high polymer network density over the complete depth of the restoration [45,46]. It also includes a patented filler (Isofiller) which functions as a shrinkage stress reliever, lessening the shrinkage force. The monomer composition of the material is a factor for the low volumetric shrinkage which causes the least microleakage [19,25]. Another benefit of this material is that its patented alkaline filler increases the release of hydroxide ions to regulate the pH value during acid attacks. There is an ongoing search for ideal restorative materials to be used after pulpotomy, in pediatric dentistry, however, a lack of evidence persists. To date, no study has been conducted where CN has been used for post-pulpotomy restoration in primary molars, to the best of our knowledge [47,48].

For the present study, ferric sulphate was used as a pulpotomy agent. For a long time, formocresol has been the most popular choice as a pulpotomy agent. Despite years of apparent successful use, formocresol has come under attack in the research and documentation in the literature which have shown formaldehyde to be toxic, mutagenic and carcinogenic [49]. Our study has used ferric sulphate because it has gained widespread attention as a pulpotomy medicament in contemporary dentistry and is classified as a preservation pulpotomy agent, since it maintains the maximum vital radicular tissue without destroying any pulp cells [50]. Ferric sulphate has been used in many previous studies as a pulpotomy agent, and has reported successful vital pulp therapies clinically as well as radiographically [31]. It has been used in comparison with formocresol and has shown equal success rates, concluding that the hemostatic and non-toxic nature of ferric sulphate makes it a promising medicament for pulpotomy procedures.

The children included in this study were between the ages of four to eight years. The age range selected was based on the time period in which there resides the greatest possibility of finding primary molars with completely formed roots. The root completion of primary molars is achieved in approximately three years, therefore the lower limit was selected as four years [51]. The resorption of the root of primary teeth begins once the hard tissue deposits in the crown, which is seven to eight years for second permanent molars, hence the upper limit for the age range was selected as eight years [51,52]. Sample size estimation was performed according to Edward and Mascha, who stated that for a sample to be able to be statistically examined, it must not be less than 30 [26].

Upon analysis of the results, there was a nonsignificant difference in the mean age as well as gender distribution between the two groups, indicating that it was a balanced sample regarding age and gender. There was also no significant difference in any of the parameters evaluated by tooth type or arch designation. It is stated in the literature that the distribution of teeth has no bearing on the scoring of the parameters, that is, maxillary or mandibular molar or first or second primary molar [53].

Different criteria have been proposed aiming to standardize the evaluation of restorative materials or operative techniques in clinical trials. US Public Health Service (USPHS) guidelines, also known as the “Ryge criteria”, is the most used criteria for evaluating restorations. These criteria are based on an assessment of biological, esthetic and functional parameters, and can be adjusted according to the needs of the user. The original criteria of color match, cavosurface marginal discoloration, anatomic form, marginal adaptation, and caries represented the five multidimensional parameters which were the major influences on the clinical judgment of a restoration’s success or failure [30]. Apart from the original five categories, there were more additions to the parameters, such as occlusion [54,55], proximal contact, gingival health, postoperative sensitivity, fracture, retention, and others. For lack of any better title, these modified lists became known as the Modified USPHS guidelines modified criteria, often called Modified Ryge criteria, and are mostly used in contemporary clinical evaluations of dental restorative materials. Modifications usually depend on the aim of the study, i.e., the types of restorations that are being compared, or are based on the study objectives. Authors from different clinical research teams do not always use the same definitions for assigning these new ratings. Thus, it has become almost a requirement to declare the categories and define the ratings as part of all publications [56].

The modified USPHS criteria were used to evaluate the two materials selected for the present study on the five parameters of marginal integrity, proximal contact, gingival health, secondary caries, and discomfort on biting. These criteria have been used for evaluating the success of post-pulpotomy restorations in several studies. One of the parameters of the USPHS criteria is marginal integrity, which is a major predictor for the long-term success of any restoration. An ideal margin for an SSC means that there is no ditching of the crown margin or subgingival extension of more than 0.5 mm, implying that there is an adaptation of the margins of the crown, with no detectable gap between the crown margin and the tooth. If there are variations in crown margin extensions, i.e., there is ditching or greater subgingival extension, this indicates that there is a gap between the tooth structure and the crown margin. In other words, the margin is deficient and non-ideal [57,58]. Whether or not the scores given for the assessment of these two different kinds of restorations can precisely be equated seems to be debatable. This can be considered a limitation of the study, comparing an intracoronal restoration with a full coverage restoration. The scoring method for the other selected parameters, i.e., proximal contact, gingival health, secondary caries and discomfort on biting, were similar for evaluating both SSCs and CN.

Marginal integrity has been extensively evaluated by researchers for a number of post-pulpotomy restorations. It was reported in a study in 2008 that marginal integrity does not statistically differ between SSCs and resin-modified glass ionomer cement at the end of 2 years [59]. Subsequently, in a study in 2011, when composites were compared with SSCs, it was reported that the marginal integrity breakdown scores were significantly higher for composites at the end of 12 months [53,60]. Another study checked the marginal seal of various restorative materials in two-surface preparations after pulpotomy. Significantly better results were obtained for resin-based restorations when compared to SSCs, glass ionomer, amalgam or zinc oxide eugenol [61]. SSCs have also been evaluated in vitro for marginal gaps, and it was seen that the specimens showed marginal discrepancy [62]. It can be concluded from our study that, as far as marginal integrity is concerned, SSC and CN restorations fare almost equally after one year. The fact that the integrity of the margins in the restoration deteriorated significantly in all of the restored teeth during the study indicates that neither of these materials can be considered ideal as far as marginal integrity is concerned.

The parameter of proximal contact depicted statistically significant differences between the two groups. There was a progressive and significantly higher breakdown of proximal contact in teeth restored with CN, and no significant breakdown in teeth restored with SSCs, at different evaluation periods. However, a study by Candice Hutcheson, comparing SSCs with composites after pulpotomy in primary molars for 12 months, found no significant differences in proximal contact between two restorations [53,60]. Another study comparing stainless SSCs with resin-modified glass ionomer cement also reported similar clinical outcomes for proximal contact of both restorations over a 24-month period [59]. Considering the present study, it can be assessed that, because of a successive and significant breakdown of CN at each follow-up, it is not an ideal restorative material for the maintenance of proximal contact. The reason why CN has not performed well clinically in maintaining proximal contact, despite having the property of higher flexural strength than other restorative materials, remains to be evaluated.

The parameter of gingival health also revealed significant differences between the two groups. Compromised gingival health was seen in teeth restored with SSCs, showing signs of gingivitis. Probing on bleeding progressed to spontaneous bleeding in this group in successive evaluation periods. However, similar effects were not seen in teeth restored with CN at different evaluation periods. Contradicting results have been reported in various studies, evaluating the effect of SSCs on gingival tissues. It was reported as early as 1974 that there is no relation between SSCs and gingival health, and no difference was seen in the gingival tissues surrounding teeth restored with SSCs and tissue surrounding uncrowned antimere [63,64]. Gingival inflammation has been cited as a frequent finding with SSCs in many studies. Compromised marginal integrity has been described as a cause of gingival inflammation in stainless steel crowns. It has been stated that a defective margin in an SSC leads to more sulcular depth, which further causes more subgingival plaque accumulation. This leads to an increase in gingival crevicular fluid leading to gingival inflammation. In a review article by Sajjanshetty [65], he concluded that when the crown is poorly adapted, its marginal integrity is reduced with chances of plaque retention and subsequent gingivitis. The results of our study are in accordance with these statements, wherein both marginal integrity and gingival status have deteriorated over the one-year study period. In the present study, the condition of the gingiva progressively deteriorated over time with SSCs as compared to CN, which implies that stainless steel crowns are not the best restorations in relation to their impact on gingival tissues.

None of the teeth in the present study in either group showed any evidence of *secondary caries* at the margins of the restoration. Illustrating similarly designed studies, a clinical trial of 24 months by M Ateih, comparing stainless steel crowns with resin modified glass ionomer cement, reported a slightly higher prevalence of secondary caries in teeth restored with the latter, illustrating that the rate of recurrent caries is lower in SSCs than in resin treated teeth [59].

Although it has been frequently reported that children generally report some discomfort after the placement of the crown, especially for SSCs, in our study, no child in either group reported discomfort on biting or any sign of discomfort at the follow-up periods. This may be attributed to the fact that occlusal disturbances in children under the age of 12 years do not cause pain or dysfunction, due to the greater adaptability of the masticatory system to occlusal abnormalities [66,67,68]. Young children appear to have an adaptable masticatory system in which changes occur quickly [69,70,71]. Similarly to our study, another study comparing stainless steel crowns with composites for a period of 24 months found no statistically significant differences between the two groups for this parameter [59]. The absence of discomfort on biting in either group indicates that these criteria cannot be used to disfavor SSCs.

The clinical parameters of pain, tenderness to percussion, swelling/sinus and tooth mobility were evaluated and showed statistically insignificant differences between the two groups at all evaluation periods. The success rates at 6- and 9-months evaluations were 100% for both groups. At 12 months, this reduced to 79.3% for CN and 86.6% for SSCs. However, the clinical outcomes were not significantly different when compared for both groups. A study by Ramazani compared SSCs, composites and bulk fill flowable composite liner in a 12-month study as post pulpotomy materials, and observed 100% success in each group with no presentation of symptoms of pain, tenderness to percussion, swelling, sinus or tooth mobility [72]. A clinical trial comparing SSC and glass ionomer-SSC evaluated the criteria of redness or swelling of the vestibular area, tenderness to percussion, tooth mobility, fistula, and abscess, in order to assess the success of the pulpotomy, and observed no clinical failure for both the treatments [73,74,75,76].

Radiographic outcomes for both treatment groups were categorized for presence or absence of radiolucency, root resorption, and widening of periodontal space in the intra-oral periapical radiograph at 12 months. Although the success rate reduced to 79.3% in the CN group and 86.6% in the SSC group, the radiographic outcomes were not statistically different. In a randomized controlled trial comparing multisurface composite versus SSCs, it was reported that all experimental and control teeth were radiographically successful in the assessment of resorption, radiolucency, and pathological changes at the end of the 12-month study period [74]. A clinical trial of six months, comparing the clinical and radiographic success of pulpotomized primary molars restored with SSCs versus glass ionomer-SSC, evaluated the radiographic criteria of periodontal ligament widening, radiolucency of furcation of apical area, and external or internal root resorption, and found no statistically significant differences in the radiographic success of both the groups [73].

This study utilized a modified version of the US Public Health Service (USPHS) guidelines [30] for evaluating restorations, since additional parameters had to be included to suit the objectives of this study. Whether the parameters used in this study were appropriate for drawing comparisons between a crown and an intracoronal restoration is a matter of debate. Moreover, pulpotomy failures can be attributed to factors such as erroneous diagnosis or the presence of previous subclinical pulpal inflammation, rather than defects in the restoration. Therefore, we suggest future studies with a longer evaluation period, comparing CN with SSCs in teeth with healthy pulp and in pulpotomised teeth, in order to draw conclusions over a wider spectrum.

## 5. Conclusions

Based on the present comparative study, conducted on 60 pulpotomised primary molars, having an initial marginal breakdown, restored with either SSCs or CN, the following conclusions can be made: significant deterioration of the marginal integrity was seen with SSCs and with CN at the end of the 12 months study period. However, no statistically significant differences were seen between either of the two restorative materials. CN showed a significantly greater proximal breakdown in successive evaluation periods than SSCs, until the end of 12 months period. SSCs showed a significant effect on gingival health, showing progressive deterioration at all evaluation periods until the end of the study period, showing signs of gingivitis and bleeding on probing. The clinical and radiographic success of teeth restored with either SSCs or CN did not differ significantly at the end of the study period.

## Figures and Tables

**Table 1:** Scoring criteria for the parameters for clinical evaluation of restorations.

Parameter	1	2	3
Marginal integrity (CN)	Explorer does not catch when drawn across the surface of the restoration towards the tooth	Explorer catches and there is visible evidence of a crevice	Explorer penetrates into a crevice that extends to the dentino–enamel junction
Marginal integrity (SSC)	0.5 mm extension of the crown into the gingival margin	1 mm extension of the crown into the gingival margin	More than 1 mm extension of the crown into the gingival margin
Proximal contact	Resistance met when passing floss	Floss passed without resistance but contact present	No contact with adjacent tooth
Gingival health	No gingival bleeding	Bleeding with probe	Spontaneous bleeding
Secondary caries	Absent	Present	
Discomfort on biting	Absent	Present	

**Table 2:** Scoring criteria for the parameters for clinical evaluation of pulpotomised molars.

Score	0	1
Pain	Absent	Present
Tenderness to percussion	Absent	Present
Swelling or sinus formation	Absent	Present
Tooth mobility	Absent	Present

**Table 3:** Scoring criteria for the parameters for radiographic evaluation of pulpotomised molars.

Score	0	1
Radiolucency in the furcation and periapical area	Absent	Present
Internal or external root resorption	Absent	Present
Widening of periodontal space	Absent	Present

**Table 4:** Statistical evaluation of three parameters for the clinical evaluation of restorations.

Clinical Finding	Group	6 Months (N = 60)			9 Months (N = 60)			12 Months (N = 59)			
		Mean	S.D.	*p* Value	Mean	S.D.	*p* Value	Mean	S.D.	*p* Value	
Marginal integrity	Group1	1.8333	0.59209	0.831	2.1667	0.59209	0.316	2.3448	0.72091	0.511	
	Group2	1.8000	0.61026		2.033	0.41384		2.2333	0.56832		
Proximal contact	Group1	1.8667	0.57135	0.001	2.1667	0.46113	0.001	2.3103	0.60376	0.001	
	Group2	1.0333	0.18257		1.0667	0.25371		1.0667	0.25371		
Gingival health	Group1	1.0667	0.25371	0.001	1.0667	0.25371	0.001	1.0345	0.18570	0.001	
	Group2	1.7333	0.52083		2.1333	0.62881		2.6333	0.49013		

Test applied: Independent sample *t*-test.

**Table 5:** Statistical evaluation of two parameters for the clinical evaluation of restorations.

Clinical Finding	6 Months (N = 60)			9 Months (N = 60)		12 Months (N = 59)	
	Group	Score	*p* Value	Score	*p* Value	Score	*p* Value
Secondary caries	Group1	0 (0.0%)	-	1 (3.3%)	0.313	1 (3.3%)	0.305
	Group2	0 (0.0%)		0 (0.0%)		0 (0.0%)	
Discomfort on biting	Group1	0 (0.0%)	-	0 (0.0%)	-	2 (6.9%)	0.143
	Group2	0 (0.0%)		0 (0.0%)		0 (0.0%)	

Test applied: chi-square test.

**Table 6:** Statistical evaluation of the clinical success of pulpotomy at 12 months.

At 12 Months		Group		*p*-Value
		I (N = 29)	II (N = 30)	
Pain	Present	1	2	0.59 (NS)
		3.3%	6.9%	
TP	Present	1	2	0.59 (NS)
		3.3%	6.9%	
Swelling	Present	2	00	0.143 (NS)
		6.9%	0.0%	
TM	Present	3	00	0.071 (NS)
		10.3%	0.0%	

TP: Tenderness to percussion; TM: Tooth Mobility; Test applied: chi-square test.

**Table 7:** Statistical evaluation of the radiographic success of pulpotomy at 12 months.

At 12 Months		Group		*p*-Value
		I	II	
Radio	Present	5	4	0.478 (NS)
		17.2%	13.3%	
RR	Present	5	4	0.478 (NS)
		17.2%	13.3%	
WPS	Present	6	4	0.343 (NS)
		20.6%	13.3%	
Total		29	30	
		100.0%	100.0%	

Test applied: chi-square test.

## Data Availability

Data can be provided on request by email by the chief researcher for academic purposes.

## Abbreviations

AAPD	American Academy of Pediatric Dentistry
IOPAR	Intra oral periapical radiograph
USPHS	United States Public Health Service Criteria
MI	Marginal Integrity
PC	Proximal Contact
GH	Gingival Health
TP	Tenderness to percussion
TM	Tooth Mobility
